# The alteration of the sensorimotor network in trigeminal neuralgia after microvascular decompression surgery: a follow-up study using independent component analysis

**DOI:** 10.3389/fphys.2025.1633028

**Published:** 2025-08-05

**Authors:** Yan Zhang, Xueju Wang, Xuefeng Wang, Gengdi Huang

**Affiliations:** ^1^ State Key Laboratory of Common Mechanism Research for Major Diseases, Institute of Basic Medical Sciences, Chinese Academy of Medical Sciences, School of Basic Medicine Peking Union Medical College, Beijing, China; ^2^ Neuroscience Center, Chinese Academy of Medical Sciences, Beijing, China; ^3^ Department of Obstetrics and Gynecology, Peking University Third Hospital, Beijing, China; ^4^ Institute of Biophysics, Chinese Academy of Sciences, Beijing, China; ^5^ Shenzhen Clinical Research Center for Mental Disorders, Shenzhen Kangning Hospital, Shenzhen Mental Health Center, Shenzhen, China

**Keywords:** trigeminal neuralgia, microvascular decompression, functional connectivity, independent component analysis, sensorimotor network

## Abstract

**Introduction:**

Trigeminal neuralgia (TN) is a chronic neuropathic pain disorder characterized by spontaneous or triggered electric shock-like facial pain. Microvascular decompression (MVD) is the most effective surgical intervention for classical TN that is refractory to medication. Recent advances in neuroimaging have enhanced visualization of the trigeminal nerve’s vascular anatomy, deepening insights into TN pathophysiology and paving the way for improved diagnostics and therapies. Resting-state functional magnetic resonance imaging (rs-fMRI) has been extensively applied in studies of TN, uncovering alterations in brain activity, functional connectivity, cortical thickness and neural networks.

**Methods:**

Independent component analysis (ICA) presents a powerful alternative for analyzing fMRI data, offering several advantages over traditional region of interests (ROIs) approaches. The sensorimotor network playing a key role in pain modulation, identifying neuroimaging differences in the sensorimotor network is crucial for detecting and intervening in TN, Forty TN patients underwent MVD surgery, with follow-up assessments conducted 6 months postoperatively and twenty-five healthy controls (HC) were recruited and scanned with resting state fMRI (rs-fMRI). Group ICA was used to identify ROIs and assessed inter-group differences in neural activity using false discovery rate (FDR) correction.

**Results:**

Compared to the HC, increased activity was observed in the right frontal operculum cortex, right insular cortex, right inferior frontal gyrus (pars opercularis), and right frontal pole in TN patients. Conversely, decreased activity was found in the right cerebellum (lobule IX) and left cerebellum (lobules VIII and IX). Compared to the pre-surgery, increased activity was found in the right precentral gyrus in the post-surgery group. Compared to the HC, long-term increased activity was still present in the right frontal operculum cortex, right insular cortex, right inferior frontal gyrus (pars opercularis), and right frontal pole despite the effectiveness of MVD surgery. In contrast, MVD significantly reduced the area of aberrant activation regions, particularly in the operculo-insular cortex, and also normalized cerebellar abnormalities.

**Discussion:**

Our study demonstrates that ICA can effectively identify distinct patterns of functional connectivity in the sensorimotor network associated with TN and MVD surgery. These regions are involved in altered pain processing, including nociceptive stimulus integration, subjective pain perception, pain chronification, and pain-related empathy. Our findings suggest promising biomarkers for TN and provide insights for developing targeted treatments.

## 1 Introduction

Trigeminal neuralgia (TN) is a chronic neuropathic disorder characterized by sudden, electric shock-like facial pain - either spontaneous or triggered - that typically affects a specific facial region and may persist between paroxysms. This disease can significantly impair quality of life, and in refractory cases, may be associated with suicidal ideation (Zakrzewska et al., 2017). The diagnosis of TN is based on three clinical criteria: (1) pain localized to one or more trigeminal nerve branches, (2) paroxysmal, electric-shock-like pain episodes, and (3) pain triggered by innocuous stimuli in the affected facial or intraoral regions. Triggered paroxysmal pain is pathognomonic of TN, occurring in 91%–99% of reported cases (Di Stefano et al., 2018; Cruccu et al., 2016; Maarbjerg et al., 2014). TN is currently classified into three distinct subtypes: classical (the most common), secondary, and idiopathic. The classical form arises from neurovascular compression (NVC) at the trigeminal nerve root entry zone (REZ), typically causing focal demyelination. NVC can be visualized using functional magnetic resonance imaging (fMRI) and 3D reconstruction. Anticonvulsants such as carbamazepine (typically 200–1,200 mg daily) and oxcarbazepine (300–1800 mg daily) are considered first-line treatments for managing paroxysmal pain in TN. Although these drugs are effective, they are associated with significant side effects and often have limited efficacy in treating persistent pain (Bendtsen et al., 2019). Microvascular decompression (MVD) has emerged as the treatment of choice for medication-refractory TN (Cruccu et al., 2016). MVD represents the most effective surgical treatment for classical TN. Postoperative outcomes demonstrate 68%–88% initial success rates (1–2 years), with 61%–80% of patients sustaining pain relief at 4–5 years follow-up (Tuleasca et al., 2014; Zakrzewska and Akram, 2011). Resting fMRI (rs-fMRI) has become an invaluable tool for examining central mechanisms, particularly in structure and mapping functional connectivity within the human brain (Biswal et al., 2010). Patients with TN show significant cortical thinning in the anterior cingulate cortex (ACC), posterior cingulate cortex (PCC), and midcingulate cortex (MCC), accompanied by extensive subcortical volume reductions (Mo et al., 2021). In addition, TN patients exhibited significantly greater cortical thickness in the contralateral primary somatosensory cortex and frontal pole compared to controls (DeSouza et al., 2013). Compared with healthy controls (HC), TN patients showed significantly altered regional homogeneity (ReHo) and fractional amplitude of low-frequency fluctuations (fALFF) in multiple brain regions, including the cerebellum, temporal lobe, cingulate cortex, putamen, limbic lobe, occipital lobe, precuneus, insula, and medial/superior frontal gyri (Yuan et al., 2018). TN patients displayed increased functional connectivity between the mPFC and left planum temporale, alongside decreased connectivity between the mPFC and left superior frontal gyrus (Wu et al., 2025). Another study revealed that patients with TN exhibited increased functional connectivity between the right insula/secondary somatosensory cortex (S2) and several key regions, including the ACC, PCC, medial prefrontal cortex (mPFC), and bilateral dorsolateral prefrontal cortex (dlPFC) (Wang et al., 2017). The sensorimotor network plays an important role in the pain modulation, as it integrates sensory input and motor output, which is crucial for bodily movements and responses to sensory stimuli. Xu et al. found that distinct patterns of functional connectivity are found in default mode network, somatosensory network, and salience network in TN (Xu et al., 2022). Disruptions in this network can be indicative of neurological disorders or conditions like TN. Costs et al. found that The TN group performed significantly worse than controls on both sensorimotor (tracking and aiming) and cognitive tasks (Coats et al., 2020). Recent fMRI studies indicate that sensorimotor impairments in low back pain (LBP) may be linked to alterations in brain function and structure (Goossens et al., 2018). Voxel-based morphometry (VBM) analyses demonstrated widespread gray matter volume (GMV) reductions throughout the brain in classical TN patients, particularly affecting the insula/secondary somatosensory cortex (S2), cingulate cortex, and multiple temporal regions (Wang et al., 2017). Zhou et al. reported that TN patients exhibited enhanced functional connectivity between the somatosensory-motor network and the dorsal attention network (Zhou et al., 2024). Tsai et al. found the activity in the sensorimotor network and default mode network was reduced in TN patients but increased following surgery (Tsai et al., 2019). Applying transcranial direct current stimulation (tDCS) over motor cortex reduces somatosensory cortex activity, which may lead to pain relief (Khodashenas et al., 2019). Currently, there remains a paucity of research investigating TN-related alterations in sensorimotor networks, as well as the impact of MVD surgery on these networks. Previous studies have analyzed blood oxygen level-dependent (BOLD) signal time series using general linear modeling (GLM), which are well-established correlates of task-evoked brain activity. However, GLM has two key limitations: it relies on a predefined experimental design matrix, and it cannot detect functional connectivity between brain regions (Kim et al., 2009; Specht and Laeng, 2011). To mitigate these limitations, we used independent component analysis (ICA), a blind-source separation method, to identify distinct clusters of brain regions with coherent hemodynamic temporal patterns without relying on prior temporal response information (e.g., an experimental design matrix). Unlike previous research that examined isolated regions, we used ICA to assess functional connectivity changes within sensorimotor networks in patients undergoing microvascular decompression surgery for classic TN, yielding potential insights for future interventions.

## 2 Materials and methods

### 2.1 Participants

This study was conducted in compliance with the Declaration of Helsinki and received ethical approval from the Federal Center of Neurosurgery’s Ethics Committee (Protocol #7, 25.05.21). This study includes raw neuroimaging data from the primary TN, collected at the Federal Neurosurgical Center, in Novosibirsk, Russia. Data were collected between 2022 and 2024. Participants were adults diagnosed with primary TN based on the International Classification of Headache Disorders (ICHD) criteria. All participants, including both patients and HC, provided written informed consent prior to study participation. Twenty-five age- and sex-matched HC were recruited via hospital advertisement systems and 40 TN patients who required MVD surgery due to pharmacological treatment failure or intolerable side effects were enrolled. Inclusion criteria were as follows:

(1) The TN group received MVD surgery and had a follow-up fMRI examination 6 months after the surgery. (2) Patients experience constant or episodic unilateral/bilateral pain in the V1, V2, and/or V3 trigeminal nerve distributions. (3) Neurovascular conflict was graded according to the M. Sindo classification system as follows: Grade 1—slight contact between the nerve and offending vessel; Grade 2—nerve distortion or displacement; and Grade 3—marked nerve indentation (Sindou et al., 2002). (4) Neuroimaging findings revealed no intracranial structural abnormalities or maxillofacial region anomalies (e.g., tumors, vascular malformations, aneurysms). (5) Right-handedness was determined based on manual asymmetry profiling. The exclusion criteria comprised: (1) History of mental disorders or neurological conditions; (2) Clinically significant cognitive impairment (Mini-Mental State Examination (MMSE) total score below the standard cutoff of 24 points) (Lecrubier et al., 1997); (3) Secondary TN (e.g., due to multiple sclerosis, brain tumors, or other intracranial lesions); (4) participants with claustrophobia; or (5) individuals with mental implants or tattoos on their neck or head.

### 2.2 Image acquisition

MRI data were acquired at the Federal Neurosurgical Center (Novosibirsk, Russia) using a 3T Philips Ingenia system (Philips Healthcare, Netherlands) with a 16-channel head-neck coil. The protocol included: 1. Functional imaging: T2*-weighted interleaved sequence (TR = 3,000 ms, TE = 30 ms, FOV = 256 × 256 mm^2^, flip angle = 90°, matrix = 80 × 80, slice thickness = 3 mm). 2. Structural imaging: High-resolution 3D sagittal T1-weighted Turbo Field Echo (TFE) sequence (TR = 6.60 ms, TE = 2.96 ms, FOV = 256 × 256 mm^2^, flip angle = 8°, matrix = 235 × 235, slice thickness = 1 mm). The total scan time was about 30 min.

### 2.3 fMRI data preprocessing and analysis

Functional connectivity analysis was conducted using the CONN-fMRI toolbox (version 22a) implemented in SPM12 (Wellcome Centre for Human Neuroimaging, London, UK; http://www.fil.ion.ucl.ac.uk/spm/). The CONN processing pipeline incorporated comprehensive preprocessing of both functional and structural MRI data, including: realignment, slice-timing correction, ART-based outlier detection, segmentation and normalization of anatomical images, functional normalization, outlier detection and smoothing (Whitfield-Gabrieli and Nieto-Castanon, 2012). All neuroimaging data underwent standard volume-based preprocessing using CONN’s default pipeline. To account for magnetic field stabilization, we discarded the first five volumes of each functional run prior to analysis. Structural images were segmented into gray matter, white matter, and cerebrospinal fluid (CSF) components and spatially normalized to the Montreal Neurological Institute (MNI) template space. Functional images were then smoothed using an 8 mm full-width at half-maximum (FWHM) Gaussian kernel. For denoising, CONN implemented component-based noise correction (CompCor) to regress out nuisance signals derived from white matter and CSF regions, along with motion parameters and other confounding variables (Behzadi et al., 2007). To minimize residual motion artifacts and physiological noise, we applied bandpass filtering (0.008–0.09 Hz), linear detrending, and scrubbing using the Artifact Detection Tool (ART). Following standard neuroimaging preprocessing protocols, we applied strict head motion exclusion criteria (translational >2.5 mm or rotational >2.5° in any direction) to minimize movement-related artifacts.

### 2.4 fMRI data processing and connectivity analysis

Group ICA was performed using CONN toolbox v22a’s default settings, identifying several robust intrinsic connectivity networks. This implementation utilized Calhoun’s group information-guided ICA (GIG-ICA) approach for group-level component extraction (Calhoun et al., 2001). Group ICA was implemented to assess connectivity changes between region-of-interest (ROI) pairs. First-level analysis conducted voxel-wise generated comprehensive functional connectivity profiles across all voxels. Local correlation analysis quantified functional segregation at each observation point through voxel-by-voxel measurements. The dimensionality was reduced to 20 independent components (ICs) based on the minimum description length criteria and previous studies (Xiang et al., 2025; Dai et al., 2023; Moser et al., 2023). Each IC represented a spatially and temporally distinct functional brain network, characterized by synchronous co-fluctuation patterns throughout the scan duration (Li et al., 2007). At the subject level, z-score normalized BOLD signals underwent dimensionality reduction via singular value decomposition (SVD), extracting 64 components per subject. These were further reduced to 20 components through group-level SVD. Group-independent networks were then identified via FastICA algorithm (fixed-point iteration with G1 hyperbolic tangent contrast function). Individual subject maps were reconstructed using GICA3 back-projection. Group-level analyses were performed within a GLM framework. For each IC, we constructed separate GLMs treating voxel-wise first-level connectivity measures as dependent variables (one per subject) and group membership as the independent variable. Voxel-wise statistical inference used multivariate parametric statistics accounting for between-subject random effects and measurement covariance. Cluster-level inference was implemented via Gaussian random field theory (GRFT), identifying contiguous voxel clusters while controlling family-wise error (FWE) rates (Whitfield-Gabrieli and Nieto-Castanon, 2012; Worsley et al., 1996). The neural relevance of these components was quantified using the Dice Similarity Coefficient (DSC), which measured spatial overlap between suprathreshold activation areas and visually identified, best-matched ICs for each functional network (Cocchi et al., 2012).

### 2.5 Statistical analyses

All statistical analyses were performed using GraphPad Prism 10.1 (GraphPad Software, USA). Demographic data normality was first verified with Shapiro-Wilk tests, parametric (Student's t-test) or non-parametric (Chi-square test) analyses was performed with appropriate *post hoc* corrections. For neuroimaging second-level analyses, between-subject contrasts were computed using a [1, −1] covariate design, with statistical significance determined by a voxel-level threshold of p < 0.001 (uncorrected), cluster-level FDR correction at p < 0.05 and a minimum cluster extent of 30 contiguous voxels. For the comparison of TN patients before and after surgery, a paired t-test was used.

## 3 Results

### 3.1 Demographic information of participants

The demographic of the study population is shown in Table 1. The data in the analysis consisted of 25 HC aged from 43 to 74 years (Mean ± SD: 56.8 ± 7.07), 40 TN patients aged from 45 to 74 years (Mean ± SD: 59.45 ± 10.38), participant details are shown in Supplementary Figure S1. The HC group consist of 10 male and 15 female, while the TN group has 18 female and 22 males. The Sindo grade in the TN patients varied from 0 to 3 (Mean ± SD: 1.68 ± 1.14). The disease duration for the TN patients was 1–30 years (Mean ± SD: 8.8 ± 6.48).

**TABLE 1:** Demographic information of participants.

Items	HC n = 25	TN n = 40	P
Age (Mean ± SD)	56.8 ± 7.07	59.45 ± 10.38	0.2661^a^
Sex (Male/Female)	10/15	18/22	0.7988^b^
Sindo grade (Mean ± SD)	0	1.67 ± 1.14	<0.0001^c^
Disease Duration	0	8.8 ± 6.48	<0.0001^c^

HC, healthy controls; TN, trigeminal neuralgia; a: Student’s t-test, b: Chi-square test, c: One sample t and Wilcoxon test analysis.

### 3.2 Independent components identification

**FIGURE 1:**
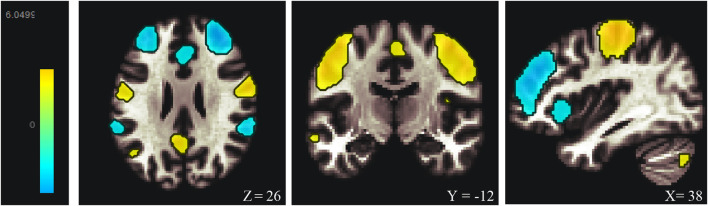
Resting-state sensorimortor networks (IC-9) generated by ICA. ICA, independent component analysis.

**FIGURE 2:**
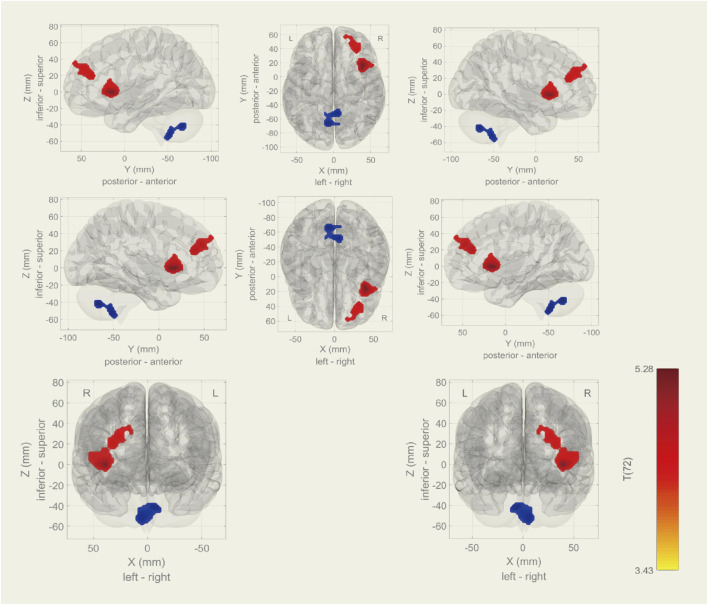
During rs-fMRI, the TN group exhibited increased activity (TN > HC) in the right frontal operculum cortex, insular cortex, inferior frontal gyrus (pars opercularis), and frontal pole. Conversely, decreased activity was found in the right cerebellum (lobule IX) and left cerebellum (lobules VIII and IX).

**FIGURE 3:**
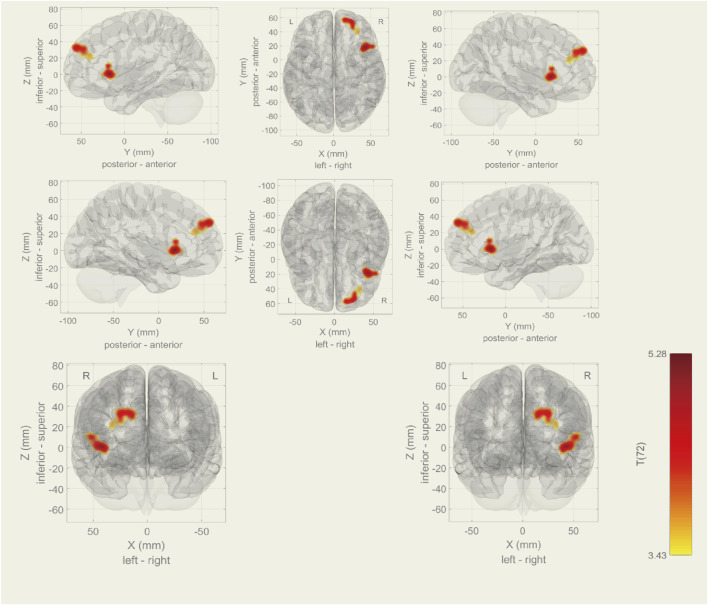
During rs-fMRI, post-surgery group exhibited increased activity (TN (Post MVD) > HC) in the right frontal pole, frontal operculum cortex, insular cortex and right inferior frontal gyrus (pars opercularis).

**FIGURE 4:**
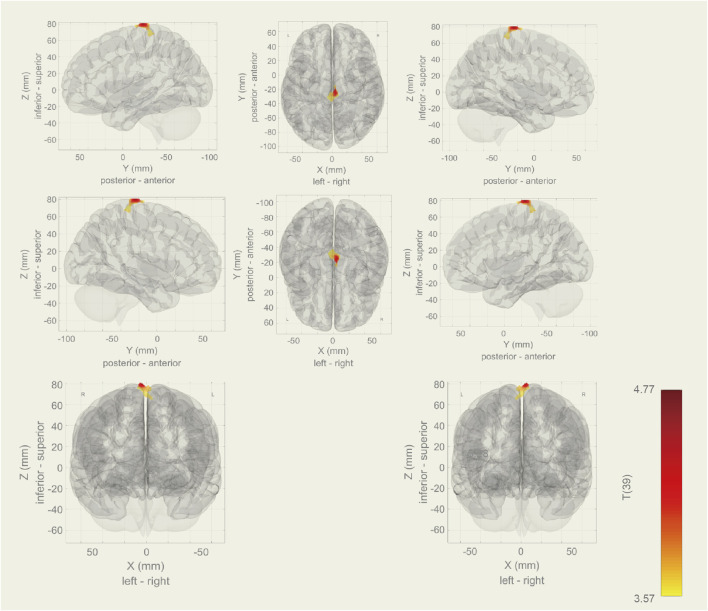
During rs-fMRI, post-surgery group exhibited increased activity in the right precentral gyrus than the pre-surgery group.

Among the 20 components, the Sensorimotor (IC-9) (r = 0.375) was selected (Figure 1). In the between subjects contrast. Enhanced activity was found in the right frontal operculum cortex, right insular cortex, right inferior frontal gyrus (pars opercularis) and right frontal pole, compared to the HC. However, decreased activity was observed in the right cerebelum (lobule IX) and the left cerebelum (lobules VIII and IX) (Figure 2; Table 2) in the TN pretreatment patients. After the MVD surgery, compared to the HC group, increased activity was observed in the right frontal pole, right insular cortex, right inferior frontal gyrus (pars opercularis), and right frontal right operculum cortex. The surgery significantly decreased the right insular cortex and operculum cortex activation area postoperatively (Figure 3; Table 3). We then assessed changes in brain activity in TN patients before and after surgery. The post-surgery group exhibited increased activity in the right precentral gyrus compared to the pre-surgery group (Figure 4). Since pretreatment TN patients’ brain activity may correlate with disease duration, we performed additional correlation analyses. However, no significant association was found between altered pretreatment brain regions and disease duration (Supplementary Figure S2).

**TABLE 2:** Rs-fMRI results (TN > HC).

MNI coordinates (x,y,z)	Total voxel size	Size P-FDR	Network	Area	ROIs (%)	Voxel size
+40 + 16 +00	488	0.000306	IC-9	Right frontal operculum cortex	63	196
				Right insular cortex	13	181
				Right inferior frontal gyrus, pars opercularis	10	67
+32 + 38 +20	324	0.004028		Right frontal pole	4	292
−6 -66–42	308	0.005288		Right cerebelum (lobule IX)	14	113
				Left cerebelum (lobules VIII)	4	71
				Left cerebelum (lobules IX)	8	67

Note: Analyses were performed using CONN, which aligns the Oxford-Harvard ROI, atlas with MNI, coordinates. Abbreviations: FDR, false discovery rate; IC, independent component; MNI, montreal neurological institute; ROI, region of interest.

**TABLE 3:** Rs-fMRI results (TN (Post-surgery) > HC).

MNI coordinates (x,y,z)	Total voxel size	Size P-FDR	Network	Area	ROIs (%)	Voxel size
+24 + 54 +34	347	0.003678	IC-9	Right frontal pole	4	330
+44 + 18 +02	276	0.011771		Right frontal operculum cortex	34	107
				Right insular cortex	6	76
				Right inferior frontal gyrus, pars opercularis	10	69

Note: Analyses were performed using CONN, which aligns the Oxford-Harvard ROI, atlas with MNI, coordinates. Abbreviations: FDR, false discovery rate; IC, independent component; MNI, montreal neurological institute; ROI, region of interest.

## 4 Discussion

This study employed ICA to examine functional connectivity changes after MVD surgery for TN. Unlike hypothesis-driven approaches, ICA is data-driven and can reveal patterns of brain dysfunction beyond the constraints of predefined experimental models. Our findings successfully revealed changes in individual functional connectivity associated with MVD surgery.

Previous studies have used ICA to identify consistent neural activity patterns across groups of individuals (Eyler et al., 2019). Compared to HC group, we found increased activity in the right frontal pole, right frontal operculum cortex, right insular cortex and right inferior frontal gyrus, pars opercularis pre-surgery of the TN group and decreased activity in the right cerebellum (lobule IX) and the left cerebellum (lobules VIII and lobules IX). The frontal pole is associated with various functions, including motor control and sensory processing (Koechlin, 2011) Changes in connectivity in this region may indicate altered neural processing related to pain. Recent neuroimaging studies have reported that pain induces both structural and functional plasticity in pain-processing brain regions, including the PFC, insula, ACC, and somatosensory cortex (Coppieters et al., 2016; Niddam et al., 2017), these changes affect sensory, emotional, and cognitive dimensions of pains. Our results are consistent with the previous study.

The insula plays a critical role in both physiological and possibly pathological pain (Garcia-Larrea, 2012; Brooks and Tracey, 2007). The insular cortex is one of the earliest brain regions activated by nociceptive stimuli. Its activation level strongly correlates with both the intensity of noxious stimuli and the subjective perception of pain (Coghill et al., 1999; Oshiro et al., 2009). The relationship between altered insular activity (Hsieh et al., 1995), structural changes (Gustin et al., 2011), and pain chronification has been increasingly recognized. In chronic LBP patients, studies have demonstrated disrupted functional connectivity of the insular with the somatosensory (Kim et al., 2019) and the medial prefrontal cortices (Baliki et al., 2011). On the other hand, pharmacological interventions for chronic pain have been shown to partially reverse these aberrant insular connectivity (Čeko et al., 2015). The observed increase in activity in the right insular cortex may indicate a neural adaptation or maladaptive changes due to chronic pain in our study. This change could reflect heightened processing of pain. In our study, we found the MVD surgery can effectively reduce the active areas in the insular cortex. However, the persistent insular activation postoperatively may contribute to the recurrence of TN in the future. Recent clinical studies have proposed the insular cortex as a potential therapeutic target for deep brain stimulation in chronic pain management. In healthy subjects, transcranial magnetic stimulation (TMS) of the posterior opercular insular cortex was found to elevate heat pain thresholds mediated by Aδ fibers (Lenoir et al., 2018). Similarly, repetitive TMS targeting the posterior insula produced significant increases in thermal pain thresholds among central neuropathic pain patients (Galhardoni et al., 2019). Notably, a study demonstrated TMS in posterior superior insula has a significant analgesic effects when applied to patients with peripheral neuropathic pain (Dongyang et al., 2021). These consistent findings across multiple pain models suggest the insular cortex may serve as a promising target for non-pharmacological pain interventions. Although our results confirm that MVD surgery is effective in reducing insular activation area, further interventions target insular cortex may be needed in the future to enhance efficacy.

The right inferior frontal gyrus (rIFG) comprises part of Broca’s area homolog, a region traditionally implicated in speech production and language processing. However, it is also involved in emotional processing, executive function, and integration of sensory information, making it relevant to pain procession and responses (Symonds et al., 2006). Recent tDCS and repetitive TMS studies suggest that the rIFG may be involved in pain-related empathy, yielding novel insights into the neurobiological substrates underlying empathy for others’ suffering (Wu et al., 2018; Li et al., 2021). Our results demonstrate that increased rIFG activity persists both pre- and post-operatively, potentially reflecting enhanced pain empathy resulting from the long-lasting pain experience in TN.

The operculum, defined as the cortical region adjacent to the insula, comprises three major subdivisions and plays well-established roles in various neurological and psychiatric disorders. This structurally complex area mediates multiple functions including sensory processing, motor control, autonomic regulation, and cognitive operations (Garcia-Larrea, 2012). The dorsal posterior insula and adjacent medial operculum are considered nociception-specific regions. Lesions in this area may predispose to neuropathic pain development, while the adjoining medial operculum mediates non-noxious thermal processing (Garcia-Larrea, 2012). Patients with chronic traumatic neck pain exhibited enhanced left pallidum-left frontal operculum connectivity compared to HC (Coppieters et al., 2021). The operculo-insular cortex plays a crucial role in pain processing. Building on this understanding, Ulf Baumgärtner and colleagues investigated somatotopic organization within this region across 11 healthy subjects using noxious heat and pinprick stimulation paradigms. Their study revealed multiple discrete pain representations within this region, likely reflecting its critical role as a multisensory integration hub that generates body site-specific emotional and behavioral responses to injury (Baumgärtner et al., 2010). Our results indicate increased activity in this brain area related neuropathic pain development in TN. MVD surgery is effective in reducing operculum activation area.

The cerebellum plays a modulatory role in pain through its communication with sensorimotor, executive, reward, and limbic functional regions. Although substantial evidence suggests that the cerebellum may be critically involved in trigeminal nociceptive pain, few studies have investigated this function. Even less is known about which specific cerebellar regions participate in trigeminal surgery. In a recent meta-analysis, Moulton and colleagues demonstrated that multiple cerebellar regions are typically activated during nociceptive processing in human, beyond this, the cerebellum contributes to pathological nociceptive sensitivity and processing in chronic pain (Moulton et al., 2010). In addition to evidence of altered activation patterns during stimulation in chronic pain, PET studies have also investigated baseline activity levels in individuals experiencing chronic pain, with some reporting sustained cerebellar blood flow changes (Hsieh et al., 1995). Beyond altered blood flow, the cerebellum in patients with chronic orofacial neuropathic pain also exhibits modified patterns of ongoing activity (Alshelh et al., 2016). In addition to observed cerebellar activation in chronic pain patients, recent neuroimaging studies have also identified abnormal functional connectivity between the cerebellum and other cortical/subcortical areas in these individuals (Simons et al., 2014). We are the first found abnormal connectivity in the right cerebellum (lobules IX) and the left cerebellum (VIII and IX) in TN. Our results indicated MVD can effectively treat this abnormality, this could be considered an important criterion for postoperative evaluation. Investigating connectivity changes pre- and post-peratively in TN is very important. They could lead to the identification of biomarkers for TN severity or potential treatment evaluation.

The precentral gyrus, also known as the primary motor cortex or Brodmann area 4, is located in the frontal lobe of the brain and is responsible for initiating and controlling voluntary movements (Banker and Tadi, 2019). The precentral gyrus also play a modulatory role in pain processing. Previous studies have identified significant group differences in functional connectivity between the right precentral gyrus and regions such as the cingulate gyrus, precuneus, and left paracentral lobule in patients with chronic shoulder pain (Wei et al., 2022). Our findings suggest that the motor cortex may serve as a key brain region in chronic pain, and that MVD can effectively enhance its activity, thereby mitigating chronic pain progression. This aligns with prior moderate-quality evidence demonstrating that high-frequency rTMS of the primary motor cortex induces significant analgesic effects in neuropathic pain (NP) and fibromyalgia (Xiong et al., 2022).

## 5 Limitations

While our findings demonstrate potential clinical relevance, this study has limitations. Most notably, the modest sample size may reduce statistical power and limit the generalizability of the results. Future research with larger samples is needed to validate these results. Second, our fMRI preprocessing used head motion thresholds of 2.5 mm translation and 2.5° rotation, which may have been insufficiently conservative. This leniency could have introduced motion-related artifacts, potentially compromising the validity of the functional connectivity results. In addition, longer-term data tracking would be beneficial to further investigate the correlation between TN recurrence post-surgery and activation in these brain regions. Notwithstanding these limitations, our findings demonstrate the potential utility of ICA for evaluating post-operative pain outcomes following MVD surgery, while providing a neurobiological foundation for developing targeted interventions.

## 6 Conclusion

Our study demonstrates that ICA can effectively identifies distinct patterns of functional connectivity associated with TN and MVD surgery. In the sensorimotor network, TN was found to increase neural activity in broad brain regions, including the right frontal operculum cortex, right insular cortex, right inferior frontal gyrus (pars opercularis), and right frontal pole. Conversely, decreased activity was found in the right cerebellum (lobule IX) and left cerebellum (lobules VIII and IX). These regions are critically involved in altered pain processing, including nociceptive stimuli integration, subjective pain perception, and pain chronification. Additionally, TN enhanced activity in areas linked to pain-related empathy while disrupting cerebellar function. Compared to the pre-surgey, increased activity was found in the right precentral gyrus in the post-surgery group. Although MVD surgery is an effective treatment, long-term hyperactivity persisted in the right frontal operculum cortex, right insular cortex, right inferior frontal gyrus (pars opercularis), and right frontal pole. However, MVD significantly reduced aberrant activation in the operculo-insular cortex and normalized cerebellar abnormalities. Our findings identify promising biomarkers for TN and provide insights for developing targeted treatments.

## Data Availability

The datasets presented in this study can be found in online repositories. The names of the repository/repositories and accession number(s) can be found in the article/Supplementary Material.

## Glossary

TN: trigeminal neuralgia
MVD: microvascular decompression
HC: healthy controls
NVC: neurovascular compression
fMRI: functional magnetic resonance imaging
rs-fMRI: resting-state functional magnetic resonance imaging
FDR: False discovery rate
REZ: root entry zone
ACC: anterior cingulate cortex
MCC: midcingulate cortex
PCC: posterior cingulate cortex
ReHo: regional homogeneity
fALFF: fractional amplitude of low-frequency fluctuations
mPFC: medial prefrontal cortex
dlPFC: dorsolateral prefrontal cortex
LBP: low back pain
VBM: voxel-based morphometry
GMV: gray matter volume
DMN: default mode networks
SN: salience network
SSN: somatosensory networks
GLM: general linear modeling
BOLD: blood oxygen level-dependent
ICA: independent component analysis
MMSE: mini-mental state examination
TFE: turbo field echo
CSF: cerebrospinal fluid
MNI: montreal neurological institute
FWHM: full-width at half-maximum
CompCor: component-based noise correction
ART: artifact detection tool
GIG-ICA: group information-guided ICA
ROIs: regions of interest
ICs: independent components
SVD: singular value decomposition
GRFT: gaussian random field theory
FWE: family-wise error
TMS: transcranial magnetic stimulation
rIFG: right inferior frontal gyrus
tDCS: transcranial direct current stimulation
