# Supplementary figures and images for: The alteration of the sensorimotor network in trigeminal neuralgia after microvascular decompression surgery: a follow-up study using independent component analysis
Source: Front Physiol. 2025 Aug 5;16:1633028. doi: 10.3389/fphys.2025.1633028 (PMC12361157; doi:10.3389/fphys.2025.1633028)

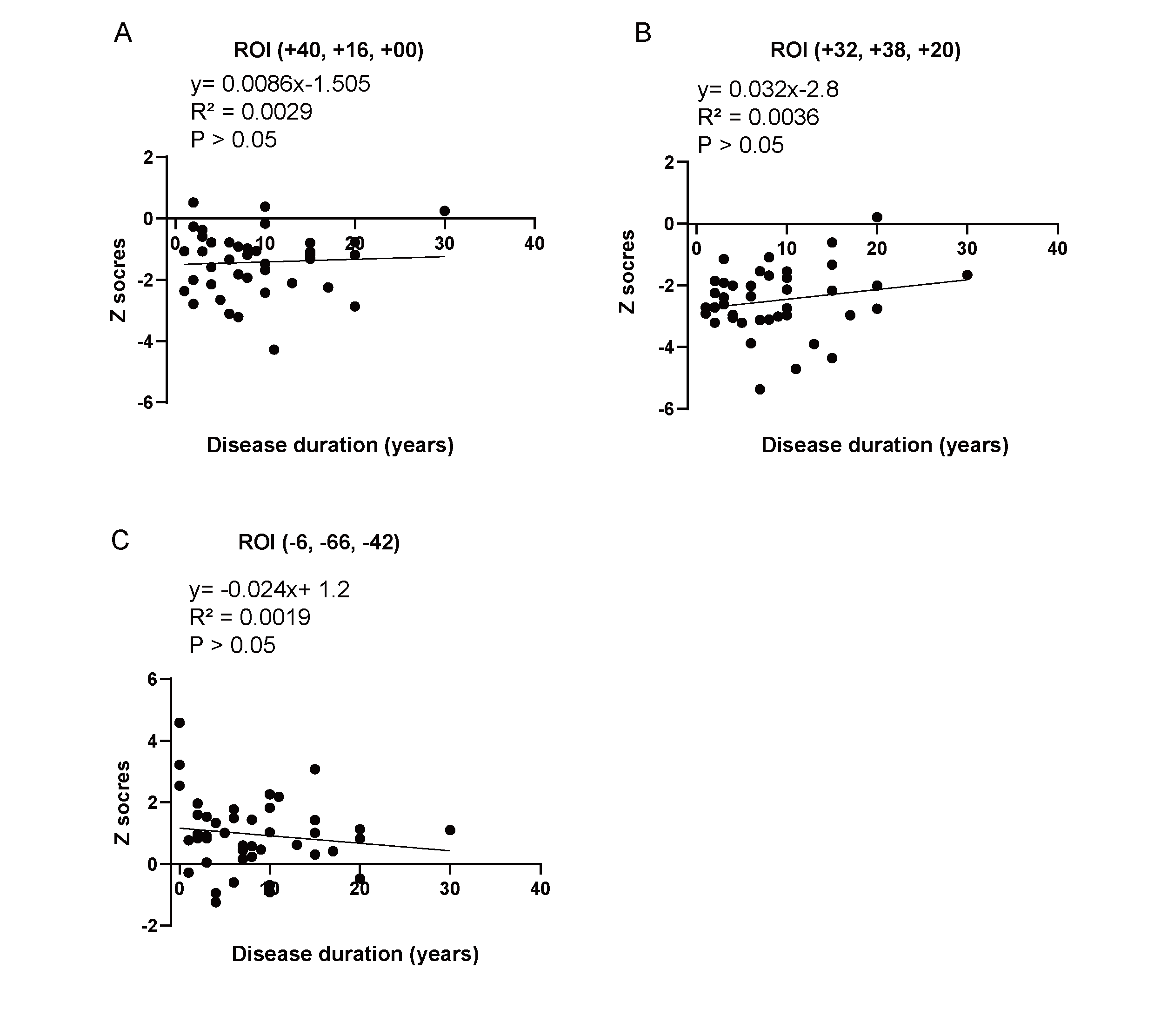

Supplement: Supplementary file 1 [file Image2.tif]

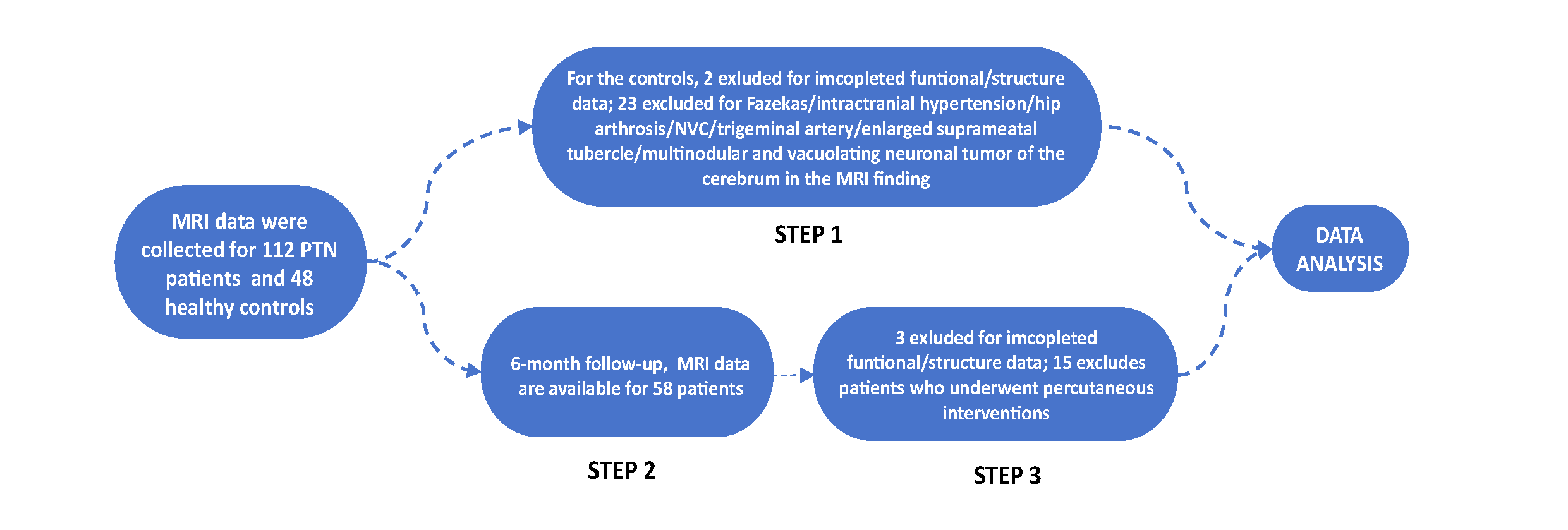

Supplement: Supplementary file 2 [file Image1.tif]
